# Is there any effect on imprinted genes H19, PEG3, and SNRPN during AOA?

**DOI:** 10.1515/med-2022-0410

**Published:** 2022-01-15

**Authors:** Rong Liang, Fang Fang, Sen Li, Xi Chen, Xiaohong Zhang, Qun Lu

**Affiliations:** Center of Reproductive Medicine, Department of Obstetrics and Gynecology, Peking University People’s Hospital, Beijing, 100044, China; Reproductive Medical Center, Department of Obstetrics and Gynecology, The Second Hospital of Guangdong Province, Guangzhou, 510317, China; Department of Obstetrics and Gynecology, Peking University People’s Hospital, Beijing, 100044, China

**Keywords:** assisted oocyte activation, intracytoplasmic sperm injection, ionophore A23187, imprinted genes, methylation

## Abstract

Assisted oocyte activation (AOA) has been proposed as an effective technique to overcome the problem of impaired fertilization after intracytoplasmic sperm injection (ICSI) but the safety of AOA remains a concern. We aimed to investigate if AOA induces imprinting effects on embryos. We used 13 cleavage embryos, nine blastocysts, and eight placentas from 15 patients. The subjects were divided into six groups by tissue type and with or without AOA. The methylation levels of imprinted genes (H19, paternally expressed gene [PEG3] and small nuclear ribonucleoprotein polypeptide N [SNRPN]) were tested by pyrosequencing. We observed different methylation levels among cleavage embryos. The variability was much more remarkable between cleavage embryos than blastocysts and placenta tissues. The methylation levels were especially higher in SNRPN and lower in the H19 gene in AOA embryos than those without AOA. No significant difference was found either among blastocysts or among placenta tissues regardless of AOA. The methylation levels of the three genes in blastocysts were very similar to those in the placenta. Compared to conventional ICSI, AOA changed imprinting methylation rates at H19 and SNRPN in cleavage embryos but not in the blastocyst stage and placenta. We recommend that blastocyst transfer should be considered for patients undergoing AOA during *in vitro* fertilization.

## 1 Introduction

The introduction and implementation of intracytoplasmic sperm injection (ICSI) have become the most successful micromanipulation procedure for treating male infertility. However, although the fertilization rate of ICSI is 70–80% [1,2], total fertilization failure still occurs in 1–3% of ICSI cycles and can reoccur in subsequent cycles [3,4,5]. Thus, although total fertilization failure after ICSI is a rare event, it may occur in the presence of a presumptively normal spermatozoon. Moreover, low fertilization (<30%) can be observed in repeated ICSI cycles for some patients [6].

After appropriate counseling, the combination of ICSI with assisted oocyte activation (AOA) is often recommended for couples dealing with total or nearly total fertilization failure after ICSI. At present, several chemical, mechanical or physical stimuli are applied to promote oocyte activation during a subsequent ICSI cycle to overcome this failed fertilization [7]. Previous studies have reported an increase in fertilization rates and utilization of cleavage stage embryos with AOA [8]. The AOA protocol is usually based on Ca^2+^ ionophores [9,10,11,12], strontium [13,14], a modified ICSI technique [15,16], or electric pulses [17,18]. Among these protocols, Ca^2+^ ionophore A23187 treatment has been widely applied in human oocyte activation [3].

During the physiological process of fertilization, the oocyte is activated by phospholipase C zeta, a sperm-borne factor [19,20,21], which induces the production of inositol-triphosphate in the ooplasm and releases calcium from the endoplasmic reticulum in an oscillatory mode [3]. Sperm-induced Ca^2+^ oscillations stimulate mitochondrial respiration and, in turn, the resulting adenosine triphosphate production is required to maintain sperm-triggered calcium waves. Nevertheless, during the AOA process, the oocyte activation with A23187 induces Ca^2+^ elevation in the form of a single transient, which is not followed by further Ca^2+^ oscillations. Beyond that, the action of A23187 can release calcium in an uncontrolled fashion from all intracellular stores, including those that would not normally be involved in the activation process. Therefore, because of the nonphysiological effect of A23187, the safety of AOA in the process of assisted reproductive technology (ART) should be carefully monitored.

The potential of calcium ionophores to support oocyte activation and achieve acceptable fertilization rates has been already tested in mice [22,23]. Moreover, retrospective studies also analyzed their oocyte activation and proposed its benefit, which resulted in healthy babies [6,10,24,25]. Yet, these studies have mainly focused on investigating the effectiveness of AOA in reproductive medicine, while few studies reported on the safety of this approach. In fact, ionophores, including A23187, exert many effects on cell homeostasis that might have a long-term effect on gene expression, some of which might be a threat and possible risk for epigenetics [26,27,28].

In this study, we evaluated the effects of AOA on imprinted genes during ART treatment. Paternal imprinted maternally expressed transcript (H19), paternally expressed gene (PEG3), and small nuclear ribonucleoprotein polypeptide N (SNRPN), which are well-studied imprinted genes, were selected for analysis. Abnormal methylation of the SNRPN gene has been reported in imprinting syndromes that with an increased prevalence in children conceived using ART [29]. H19 is a paternally methylated imprinted gene; its alterations have been described in placentas from ART pregnancy [30]. The loss of PEG3 imprinted methylation has been observed in mouse blastocysts derived from ART [31]. By using the donated embryos and placenta tissue, we compared the methylation status of differentially methylated regions (DMRs) of these three key imprinted genes between patients with and without AOA.

## 2 Methods

### 2.1 Ethical approval

The study was approved by the Ethical Committee of Peking University People’s Hospital (approval number 2011-67). All the patients delivered a healthy baby after ART treatment. Surplus embryos and placenta tissues were donated for research with written consent (Table A1).

### 2.2 IVF-ET treatment protocols and artificial oocyte activation

The women who were offered AOA had at least one total or nearly total fertilization failure after ICSI in previous cycles, and one couple with globozoospermia was offered AOA on half oocytes and conventional ICSI on the remaining half oocytes. The patients who underwent conventional ICSI treatment (nonassisted oocyte activation [NOA]) were used as controls; the two groups were matched by age.

The individual stimulation protocols for *in vitro* fertilization & embryo transfer (IVF-ET) were determined according to the age of the patient and the ovarian reserve status, including the antral follicle count, basal levels of follicle-stimulating hormone (FSH), luteinizing hormone, and estradiol (E_2_). Most women underwent the long luteal downregulation protocol. Briefly, 1.25 mg of gonadotropin-releasing hormone agonist (GnRHa, Diphereline^®^, Beaufour-Ipsen Pharmaceuticals Ltd., Paris, France) was injected on menstrual day 21. The initial gonadotropin dose was based on the physician’s discretion but always contained an amount of rFSH, supplemented with at least one ampoule (75 IU) of human menopausal gonadotropin. For the flare-up agonist stimulation, a dose of rFSH along with a fixed dose of GnRHa (0.1 mg/day, triptorelin, Ferring, Saint-Prex, Switzerland) was administered beginning on menstrual day 2. Human chorionic gonadotropin (HCG, 10,000 IU, Lizhu Ltd., Guangdong, China) was administered when at least two follicles were 18 mm in diameter. Oocytes were retrieved by transvaginal ultrasound-guided follicular aspiration 36 h later. Oocytes were fertilized using ICSI.

For artificial oocyte activation, 30 min after ICSI, oocytes were incubated in a culture medium containing 10 mM calcium ionophore A23187 (Sigma) for 10 min at 37°C and 6% CO_2_. The oocytes were then extensively washed and placed in a culture medium (G-1; Vitrolife) in the incubator under 6% CO_2_, 5% O_2_, and 89% N_2_.

The fertilization results (two pronuclei, 2PN) were assessed 16–20 h after insemination. High-quality transferred or frozen embryos were defined as embryos developed from normally fertilized eggs, with no more than 20% fragmentation, no multinucleation, and 7–8 blastomeres, 72 h after egg retrieval. Blastocysts scored as Gardner’s classification were transferred or frozen if they reached at least third-stage expansion with A or B for inner cell mass (ICM) or trophectoderm. One or two embryos per patient were transferred on the third or fifth day after oocyte retrieval. Surplus embryos were frozen by vitrification procedure (KITAZATO). After the patients agreed and decided to donate, frozen embryos were thawed, and embryo quality was evaluated. Next, samples were frozen in nitrogen for DNA methylation analyses. Donated placenta tissue was collected within 30 min after delivery and frozen in nitrogen.

### 2.3 Pyrosequencing

Genomic DNA from a single embryo or placenta was isolated with the DNeasy Blood and Tissue Kit (Qiagen). Subsequent bisulfite conversion was performed using an EpiTect Bisulfite Kit (Qiagen), following the manufacturer’s guidelines. The primers for a polymerase chain reaction and pyrosequencing (Table 1) were designed using PyroMark Assay Design 2.0 software (Qiagen). Polymerase chain reaction amplification for H19, SNRPN, and PEG3 was performed with an initial denaturation step at 95°C for 2 min, 36 cycles at 95°C for 15 s, primer-specific annealing temperature for 15 s, and 72°C for 15 s; a final extension step was completed at 72°C for 7 min. The amplification reaction was carried out in a final volume of 50 µL, containing 2 µL of DNA, 12.5 µL of ready-mix (KAPA 2 G Robust HS ReadyMix), 1 µL of each primer (50 pM/µL) and 8.5 µL H_2_O. Pyrosequencing of the PCR fragments was performed on a Pyro-Mark Q96 ID pyrosequencing system (Qiagen). Pyro Q-CpG software (Qiagen) was used for data analysis.

**Table 1:** Primers used for H19, PG3, and SNRPN methylation analysis

Imprinted gene	Primer sequence	Amplicon length (base pair)	Number of CpGs site
H19	Forward	253	6
	AGGGTTTTTGGTAGGTATAGAG		
	Reverse		
	CCTATTCCCAAATAACCCC		
	Sequencing		
	GTGGAATAGGAAGTGGT		
PEG3	Forward	154	4
	GGTGTAGAAGTTTGGGTAGTT		
	Reverse		
	ACTCACCTCACCTCAATACTAC		
	Sequencing		
	GTTTATTTTGGGTTGGT		
SNRPN	Forward	220	7
	GGGAGGGAGTTGGGATTTTTGTA		
	Reverse		
	AAACCACCCACACAACTAACCTTAC		
	Sequencing		
	GGAGTTGGGATTTTTGTAT		

### 2.4 Statistical analyses

Statistical analyses were performed with SPSS version 17.0. For each imprinted gene, the difference in DNA methylation between embryos was assessed by a one-way ANOVA test followed by Turkey multiple comparison tests. If the data sets were not normally distributed, the nonparametric Mann–Whitney *U* test was used to assess between-group differences. A *P*-value of <0.05 was considered statistically significant.

## 3 Results

In order to evaluate the possible impact of AOA on epigenetics, we quantified DNA methylation of three imprinted genes (PEG3, SNPRN, and H19) using pyrosequencing on cleavage embryos, blastocysts, and placenta. A total of 13 cleavage embryos, nine blastocysts, and eight placentas were included in this study. As highlighted in Table 2, four cleavage embryos and four blastocysts were derived from AOA, and nine cleavage embryos and five blastocysts from NOA (conventional ICSI). All these embryos came from the same cycle, in which the patients had one or two healthy babies after treatment. In addition to these embryos, placentas were also collected from eight patients; three underwent AOA and five underwent NOA. There were no significant differences between the two groups with regard to women’s age, body mass index, treatment protocol, oocytes, and high-quality embryos (Table A2).

**Table 2:** Summary of different groups for DNA methylation analysis

Group	Tissue type	Number	Method of fertilization	AOA	Live birth (patient who donated)
AOA-C	D3 cleavage embryo	4	ICSI	Yes	Yes
NOA-C	D3 cleavage embryo	9	ICSI	No	Yes
AOA-B	Blastocyst	4	ICSI	Yes	Yes
NOA-B	Blastocyst	5	ICSI	No	Yes
AOA-P	Placenta	3	ICSI	Yes	Yes
NOA-P	Placenta	5	ICSI	No	Yes

**Figure 1:**
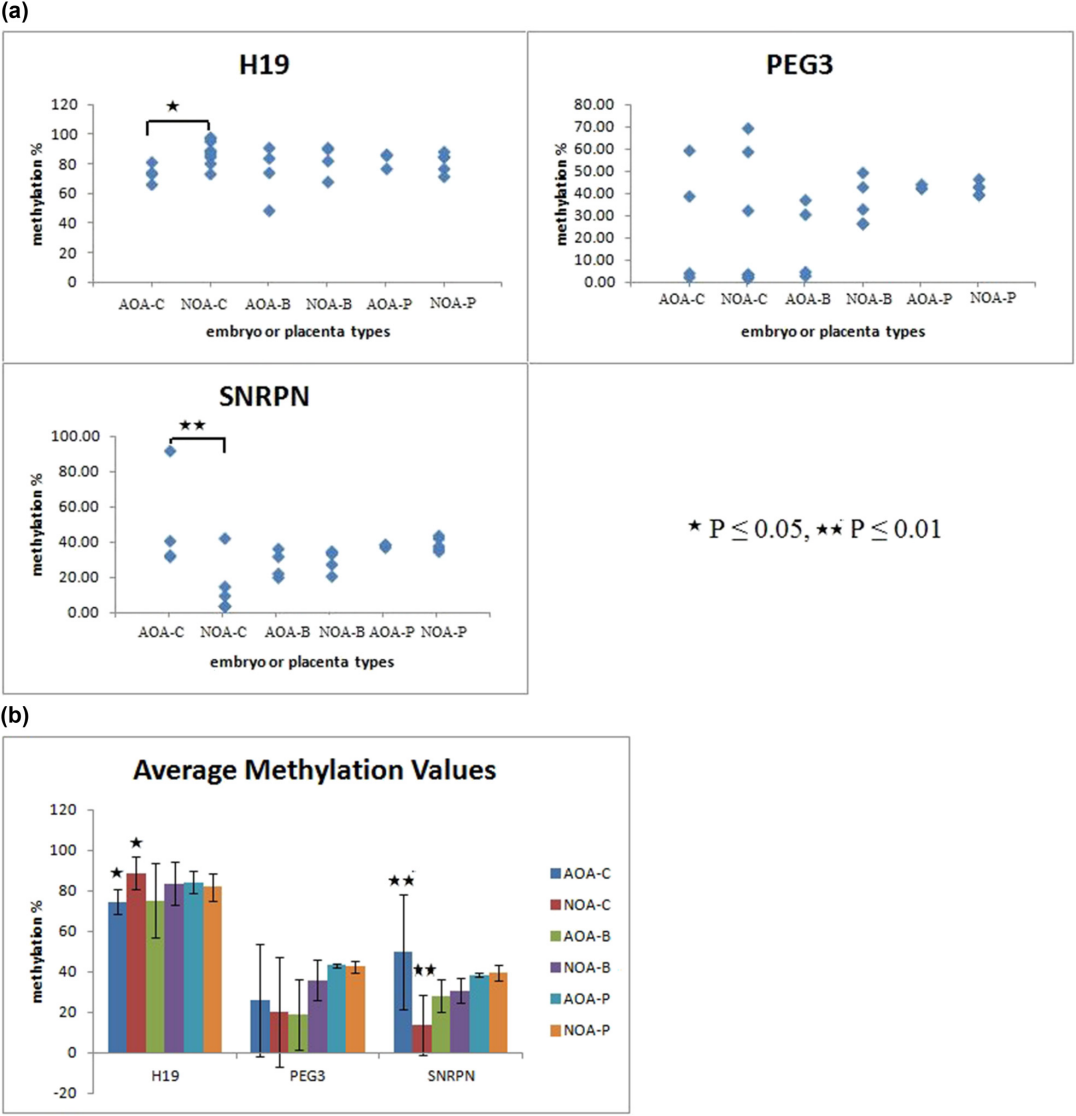
Pyrosequencing analysis of DNA methylation at three imprinted genes for different types of embryos or placentas. (a) The global methylation percentage in the methylated allele of H19, PEG3, and SNRPN for different embryos or placentas. Each dot corresponds to an embryo or a placenta. (b) Average methylation values plotted with standard deviation values for a different group. For genes H19 and SNRPN, the difference between AOA-C and NOA-C was significant (*P* ≤ 0.05 and *P* ≤ 0.001).

The average methylation levels of H19, PEG3, and SNRPN for each embryo or placenta are shown in Figure 1. First, we compared the methylation levels in cleavage embryos (AOA-C versus NOA-C), blastocysts (AOA-B versus NOA-B), and placentas (AOA-P verse NOA-P) between groups. The greatest range in methylation values occurred in the groups AOA-C and NOA-C, especially for PEG3 (2.5–59.25% in the AOA-C group and 2.0–59% in the NOA-C group) and SNRPN (32.18–92% in the AOA-C group and 4.14–42.57% in NOA-C group); for H19 and SNRPN, the difference in DNA methylation between AOA-C and NOA-C groups was significant (*P*-value ≤0.05 for H19 and *P*-value ≤0.001 for SNRPN). For the gene PEG3, no significant difference was observed between AOA-C and NOA-C groups. More importantly, in the blastocysts and placentas the variance of three genes was no difference between two groups.

**Figure 2:**
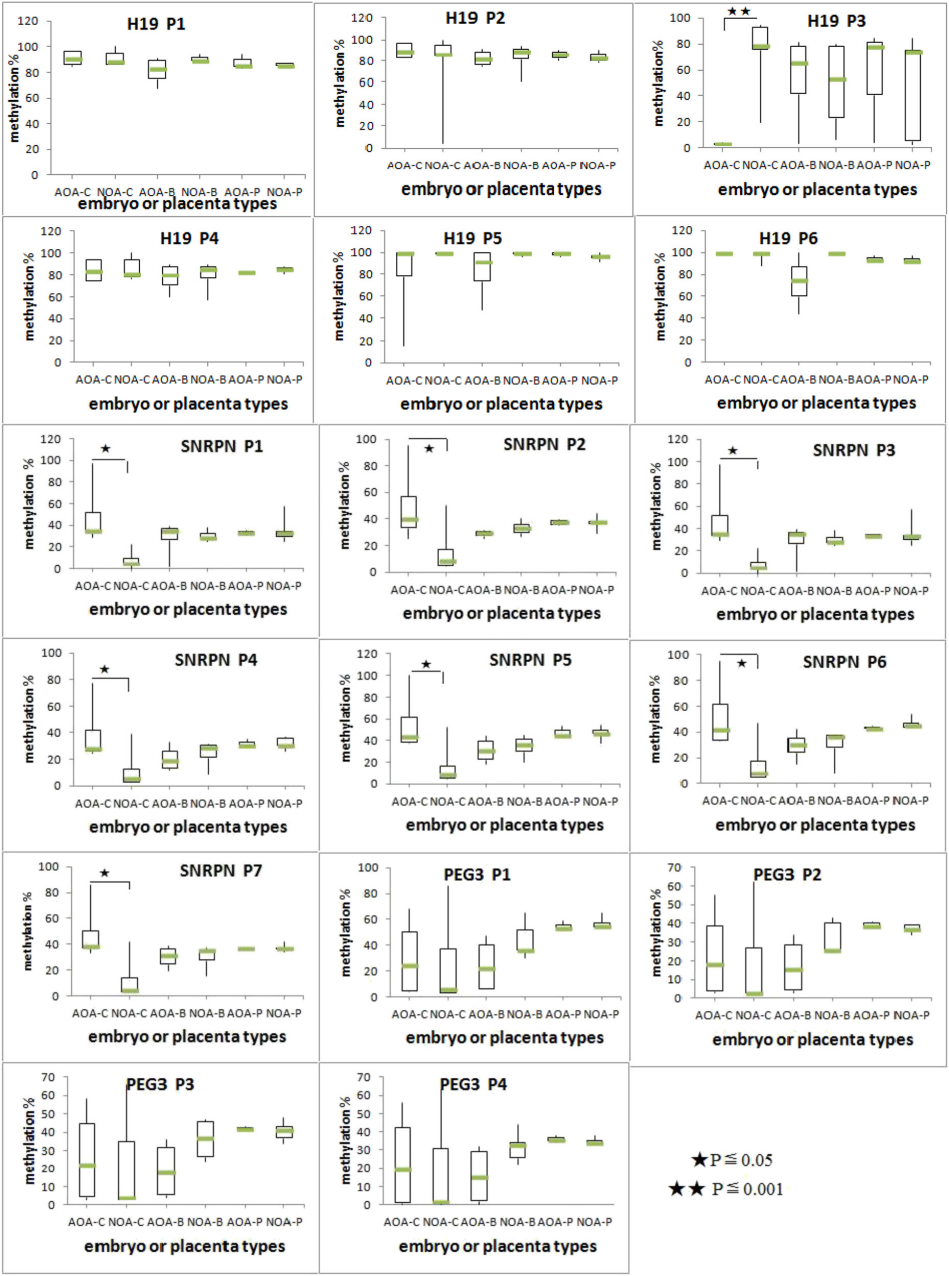
The methylation profile of methylated alleles of H19, SNRPR, and PEG3 for a different group. Boxplot diagrams present the methylation percentage of all CpG sites across H19 (H19 P1-P6), SNRPR (SNRPN P1-7), and (PEG3 P1-P4) in six groups (AOA-C, NOA-C, AOA-B, NOA-B, AOA-P, and NOA-P). The box represents the interquartile range, which contains 50% of values. The whiskers are lines that extend from the box to the highest and lowest levels. A line across the box indicates the median value for each group. Statistical significance values are as follows: *P ≤ 0.05 and **P ≤ 0.001.

The methylation profiles of individual imprinted alleles for H19, SNRPN, and PEG3 are shown in Figure 2. The number of DMR-associated CpG dinucleotides analyzed for each gene is as follows: H19 (*n* = 6), SNRPN (*n* = 7), and PEG3 (*n* = 4). The DNA methylation status of each individual CpG was then determined by pyrosequencing. For the gene SNRPN, the comparison between AOA-C and NOA-C groups revealed a significant difference; the methylation levels of all the 7 CpG sites were significantly higher in cleavage embryos of AOA than those of NOA (*P* < 0.05). For the gene H19, methylation values on the third site were significantly different between the cleavage embryos, which were lower in the AOA-C group than in the NOA group (3.45 ± 0.47% versus 76.33 ± 22.69%, *P* < 0.05). For PEG3, although there was a great deviation on cleavage embryos, no significant difference was observed between AOA-C and NOA-C groups (*P* > 0.05). Notably, for all these CpG sites of three imprinted genes, the methylation levels showed no significant difference in blastocysts with or without AOA (AOA-B versus NOA-B, *P* > 0.05). Similar data were obtained when comparing the methylation variance between placenta in the two groups (AOA-P versus NON-P).

**Figure 3:**
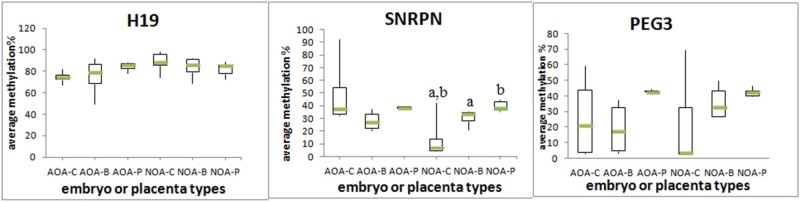
Boxplot diagrams showing the average methylation percentage of H19, SNRPR, and PEG3 in six groups (AOA-C, AOA-B, AOA-P, NOA-C, NOA-B, and NOA-P). Comparison results between groups without activation and between groups with activation were separately conducted. Boxplots with the same superscripts are significantly different (*P* value <0.05).

The development trend of the three imprinted genes from cleavage embryos, the blastocyst to the placenta is shown in Figure 3. For the three genes, the relative methylation level had a similar kinetic trend from the blastocyst stage to the placenta, both in the AOA group and NOA group. The methylation level in the blastocyst and placenta was very close in both groups. When comparing among AOA-C, AOA-B, and AOA-P or comparing among NOA-C, NOA-B, and NOA-P, there was no significant variance for the methylation levels of genes H19 and PEG3 (*P* > 0.05). The results were different for the gene SNRPN. Comparison across the groups without activation revealed that a significant difference existed between NOA-C and NOA-B and NOA-C and NOA-P. The methylation level of SNRPN in cleavage embryos was much lower than those in blastocysts and placentas. However, there were no significant differences in any of the performed comparisons across the group with activation.

## 4 Discussion

AOA is an effective method for avoiding total fertilization failure. It can repair defective activation and improve ICSI outcomes. Clinical trials suggested that birth outcomes and health for children from artificial oocyte activation techniques are comparable to those children conceived by conventional ICSI [32] and that the developmental outcomes of children 3–10 years born after AOA are within expected ranges [25]. Yet, the number of live births after AOA is still low. Nonetheless, the sole concept of using live birth as the end-point for successful IVF is somewhat controversial [33]. Although the use of calcimycin or ionomycin, which can increase membrane permeability to extracellular Ca^2+^, had not been linked with any deleterious effects and did not cause chromosomal abnormalities [34,35], their safety and potential long-term effects during embryogenesis are still unknown. Recently, Chen et al. showed that a high concentration of ionomycin increased DNA damage and decreased mouse blastocyst formation [36]. This study indicated that the improper application of AOA may have adverse effects on pre-implantation embryo development.

Biochemical processes during artificial oocyte activation are well-investigated [37]; yet, most data come from preclinical studies. Because gametogenesis and embryogenesis exhibit considerable species differences, particularly between humans and rodents, experimental findings in animal models cannot directly and accurately reflect the real situation in humans. Knowing that ionophores can affect cell homeostasis and, in turn, have long-term effects on gene expression, concerns have been raised regarding the interference of AOA in the epigenetic quality of the oocyte and embryo. Sill, evidence reporting epigenetic effects of AOA on humans is limited. ARTs and infertility may also be associated with epigenetic disorders such as the disruption of genomic imprinting [38,39,40]. Thus, further investigation is needed to investigate whether oocyte activation may cause epigenetic modifications as assumed for *in vitro* operation or culture media.

In many studies, imprinted genes were used as a model for studying ART-induced epigenetic changes in oocytes [41,42,43,44,45,46]. In this study, surplus embryos donated by patients who underwent ART were used to investigate the impact of oocyte activation on the methylation of imprinted genes. Our data suggested that the methylation levels for all three genes (H19, PEG3, and SNRPN) were altered in embryos obtained by AOA [31]. The methylation levels of H19 and SNRPN in cleavage embryos obtained by activation were significantly different from embryos obtained without activation. The methylation level of SNRPN was much higher, and the methylation level of H19 was much lower in a group with activation, while no difference was observed for PEG3. Based on these results, it was not possible to assess which level was exactly right as we did not have the information on the epigenetic variation for embryos from fertile couples. Yet, these data suggested that AOA might impact certain imprinted genes compared to traditional ICSI. Our results support the hypothesis that the combination of ARTs could induce more epimutations in the embryos as was previously suggested [40,47].

Apart from being accurately established during gametogenesis, genome-wide changes in DNA methylation may occur during the pre-implantation period. The dramatic DNA demethylation occurs from fertilization and the two-cell stage human embryo and reaches the lowest DNA methylation at the blastocyst’s ICM. Greater global demethylation and DNA remethylation changes make the embryo more susceptible to disturbances [46,48,49]. In this study, most of the obvious changes were observed in cleavage embryos, suggesting that cleavage embryos were more vulnerable to AOA. We also noticed that the methylation values of imprinted genes were more stable in the blastocyst and placenta either from the activation group or from traditional ICSI. Also, the methylation level in the blastocyst was very close to that in the placenta. Thus, it seems that the influence of AOA on imprinted genes tends to be stable after the embryos develop to blastocyst. Because the blastocyst in this study could not be the same one that developed from cleavage embryos involved in the study, it was not easy to ascertain whether the process of self-adjustment to the influence of AOA during the embryo development does exist. However, based on the similar methylation states of blastocysts and placentas in either the AOA group or NOA group, it seems that if the embryo survived and developed to a blastocyst, the impact of AOA on future generations might be reduced to a minimum.

An increased incidence of imprinting disorder after ART has been described in humans. For example, ART-associated Angelman syndrome associated with hypomethylation at the SNRPN imprinting control region had been previously reported [50,51,52]. Previous studies in mice showed that ARTs might result in significantly lower global methylation and a higher number of abnormal alleles for maternal SNRPN in embryos when compared with embryos developed *in vivo*. Interestingly, our results showed that the methylation level of SNRPN was significantly higher in embryos from AOA, complicating the study of the individual effect of oocyte activation. It seemed that the imprinted gene SNRPN was a very sensitive epigenetic mark to survey the effects of ARTs on epigenetics. We also noticed that some individual embryos presented the lowest methylation levels for all three genes. Whether this might be related to their reduced developmental competence should be further investigated.

This study has some limitations. First, the number of samples was relatively small. Our results only elucidated the epigenetic effect of AOA on some specific embryos donated by patients. Second, we only focused on the three well-selected imprinted genes; more imprinted genes should be examined in future studies. Third, the embryos and placentas came from different couples. Because the imprinted genes can show considerable methylation variation among normal individuals, further experiments need to be performed by increasing the sample size, and the epigenetic alteration of baby born from AOA should be specified. Although we found that AOA had some effect on imprinted genes, especially for SNRPN, the functional consequences of methylation changes on the imprinted gene remain to be elucidated. Before that, AOA still needs to be considered as experimental [6,25]; its application requires thorough consultation with the patient and should only be done if correct indications are present.

## Abbreviations

AOA: assisted oocyte activation
ARTs: assisted reproductive technologies
DMRs: differentially methylated regions
E_2_
: estradiol
FSH: follicle stimulating hormone
HMG: human menopausal gonadotropin
ICSI: intracytoplasmic sperm injection
IP3: inositol-triphosphate
LH: luteinizing hormone
NOA: nonassisted oocyte activation
PEG3: paternally expressed gene
SNRPN: small nuclear ribonucleoprotein polypeptide N

## Appendix

**Table A1:** Summary of patients for donation of embryos and placenta

Group	Patients	Age	Indication for IVF	History of total or nearly total fertilzation failure after ICSI	Tissue type	Number	Live birth
AOA-C	1	30	Nonobstructive-azoospermia	1	D3 cleavage embryo	2	A healthy female baby
AOA-P					Placenta	1	
AOA-C	2	20	Nonobstructive-azoospermia	1	D3 cleavage embryo	2	A healthy female and a male baby
AOA-B					Blastocyst	1	
AOA-B	3	32	Globozospermia	No	Blastocyst	2	A healthy male baby
AOA-B	4	30	Nonobstructive-azoospermia	2	Blastocyst	1	A healthy female baby
AOA-P	5	36	Nonobstructive-azoospermia	3	Placenta	1	A healthy male baby
AOA-P	6	31	Severe oligoasthenospermia	2	Placenta	1	A healthy male baby
NOA-C	1	34	Severe oligozoospermia	No	D3 cleavage embryo	4	A healthy male baby
NOA-C	2	34	Severe teratozoospermia	No	D3 cleavage embryo	5	Two healthy female babies
NOA-B	3	31	Severe teratozoospermia	No	Blastocyst	2	A healthy female baby and a male baby
NOA-B	4	32	Globozospermia	No	Blastocyst	2	A healthy male baby
NOA-P	5	28	Obstructive-azoospermia	No	Blastocyst	1	A healthy male baby
NOA-P	6	32	Oligozoospermia	No	Placenta	1	A healthy female baby
NOA-P	7	31	Severe oligozoospermia	No	Placenta	1	A healthy female baby
NOA-P	8	28	Severe oligozoospermia	No	Placenta	1	A healthy male baby
NOA-P	9	30	Obstructive-azoospermia	No	Placenta	1	A healthy female baby
NOA-P	10	33	Nonobstructive-azoospermia	No	Placenta	1	A healthy male baby

**Table A2:** Characteristics and Ovarian hyperstimulation parameters of AOA and conventional ICSI undergoing IVF-ET

Characteristics	Controls (*n* = 9)	Cases (*n* = 6)	*p*
Age (year)	31.30 ± 2.16	29.83 ± 5.31	0.445
BMI (kg/m^2^)	22.00 ± 1.41	22.09 ± 2.10	0.917
Fasting glucose	4.97 ± 0.45	5.06 ± 0.46	0.678
Fasting insulin	7.68 ± 1.07	8.00 ± 0.82	0.543
Insulin resistance	1.70 ± 0.32	1.79 ± 0.14	0.442
Primary infertility	9 (100%)	6 (100%)	1
Treatment protocol			0.287
I	5 (55.56%)	5 (83.33%)	
II	3 (33.33%)	0 (0)	
III	1 (11.11%)	1 (16.67%)	
Basal FSH (IU/L)	7.27 ± 0.70	7.78 ± 1.68	0.403
Basal LH (IU/L)	4.84 ± 0.53	4.73 ± 1.26	0.815
Basal E_2_ (pg/L)	38.30 ± 6.96	47.33 ± 10.91	0.06
Antral follicle count (*n*)	12.80 ± 0.92	12.17 ± 5.38	0.716
Days of stimulation	9.90 ± 1.79	11.33 ± 2.16	0.173
Total dose of gonadotropins (U)	2212.50 ± 710.85	2875.00 ± 1144.00	0.171
Number of follicles >13 mm	16.50 ± 9.52	15.67 ± 7.50	0.858
Number of oocytes retrieved	17.80 ± 9.51	18.83 ± 10.79	0.941
Number of MII oocytes (*n*)	13.90 ± 4.33	13.67 ± 8.12	0.796
Number of 2PN (*n*)	9.90 ± 5.30	9.17 ± 5.38	0.856
Number of high-quality embryos	4.50 ± 2.65	3.40 ± 1.95	0.494
